# Elderly people with hearing loss and cognitive decline: speech perception performance in noise

**DOI:** 10.1590/2317-1782/20242023094en

**Published:** 2024-06-14

**Authors:** Maria Julia Ferreira Cardoso, Kátia de Freitas Alvarenga, Maria de Lourdes Merighi Tabaquim, Tatiana de Andrade Lopes, Orozimbo Alves Costa, Lilian Cássia Bórnia Jacob

**Affiliations:** 1 Programa de Pós-graduação em Fonoaudiologia, Faculdade de Odontologia de Bauru – FOB, Universidade de São Paulo – USP - Bauru (SP), Brasil.; 2 Departamento de Fonoaudiologia, Faculdade de Odontologia de Bauru – FOB, Universidade de São Paulo – USP - Bauru (SP), Brasil.

**Keywords:** Elderly, Hearing Loss, Speech Perception, Cognition, Cognitive Impairment

## Abstract

**Purpose:**

To verify the influence of verbal intellectual-cognitive skills on speech perception in noise, in elderly with sensorineural hearing loss, considering education, age, and degree of hearing loss.

**Methods:**

36 elderly between 60 and 89 years old with bilateral sensorineural hearing loss participated in the study. After psychological assessment using the Wechsler Intelligence Scale for Adults (WAIS-III), they were grouped into (GI) 24 elderly without cognitive alteration and (GII) 12 elderly with risk of cognitive alteration. They underwent otorhinolaryngological assessment, audiological interview, pure tone audiometry, and assessment of speech perception in noise using the Hearing in Noise Test (HINT-Brazil). The Mann-Whitney U statistical test compared the results between the groups, and the Spearman correlation verified the variable's age, degree of hearing loss, and level of education.

**Results:**

There was no difference between the groups in the ability to perceive speech in noise, except in the noise on the left condition, in which GII showed better performance in HINT-Brazil. The degree of hearing loss and level of education influenced the perception of speech in noise. The level of education was correlated with the WAIS-III results.

**Conclusion:**

The decline in verbal intellectual-cognitive skills did not affect speech perception of noise in the elderly with hearing loss. The degree of hearing loss and level of education influenced the performance of the elderly in the speech perception test in noise. Performance in verbal cognitive skills varied according to the level of education.

## INTRODUCTION

Age-related hearing loss is one of the most prevalent chronic health conditions in old age, resulting from the cumulative effects of aging on the auditory system^(1)^. The characteristics of age-related hearing loss are progressive, bilateral sensorineural type, symmetrical, and descending audiometric configuration with greater involvement in high frequencies^(2)^.

With the increase in life expectancy in Brazil^(3)^, hearing health services receive more and more elderly people with complaints regarding audibility and speech understanding in unfavorable environments. The increase in demand is related to the search for the acquisition of hearing aids, aiming to alleviate hearing difficulties and improve the main complaint, which is characterized by difficulty in speech perception, especially in the presence of competitive noise^(4,5)^.

This difficulty is not only justified by changes in the peripheral auditory system but also by changes in the central auditory nervous system^(6,7)^. Parallel to the process of hearing changes related to aging, a decline in intellectual abilities and cognitive functions may occur, which has been associated with greater changes in speech perception in competitive noise^(5)^, that is, there is evidence of an association between worse performance in the speech perception test in competitive noise and the decline of cognitive functions^(8)^.

However, there are different hypotheses regarding the etiological mechanisms involved with age-related hearing loss and decline in cognitive functions^(1)^: cognitive load hypothesis: it is theorized that hearing loss leads to the deterioration of auditory signals, with greater cognitive resources being recruited for auditory perceptual processing and the diversion of other cognitive resources to listening with listening effort, causing a depletion of the cognitive reserve^(2)^; common cause hypothesis: states that both hearing loss and changes in cognitive functions result from a common neurodegenerative process in the aging brain^(3)^; cascade hypothesis: explains that peripheral hearing loss affects brain structure directly through degraded sensory information, that is, age-related hearing loss is associated with smaller brain volume and accelerated rates of brain atrophy; and^(4)^ overdiagnosis or precursor hypothesis: hearing loss impacts performance on certain neuropsychological tests, i.e., because verbal instructions or tasks that rely considerably on hearing are used during cognitive tests, individuals with hearing loss may be at a disadvantage^(9,10)^. Faced with this multifaceted reality, the complex relationship between aging, hearing loss, cognition, and speech perception in noise emerges.

Regardless of the theory that underpins this relationship, hearing loss was considered a modifiable risk factor for changes in cognitive function and its implications, representing the highest proportion of attributable threat in comparison to other variables analyzed such as diabetes, obesity, hypertension, among others^(11,12)^.

On the other hand, there are controversial findings in the literature, since studies did not find a relationship between the degree of hearing loss and cognition^(12-14)^ and did not observe a relationship between cognition and the ability to perceive speech in noise^(15)^. However, the meta-analysis of a systematic review showed that the cognitive functions of inhibitory control, working memory, episodic memory, and processing speed are important for the ability to perceive speech in noise^(8)^. Therefore, they are current themes in the study of hearing loss related to aging.

Another variable considered important in studies involving cognition is education^(16)^, as even in individuals who do not present changes in cognitive function, the lower the level of education, the lower the score on cognitive function tests. Therefore, its influence on cognitive status^(16,17)^ must be considered in studies including the assessment of cognitive abilities.

There is a lack of studies in the national literature that investigate the association between age-related hearing loss, changes in cognitive functions and difficulty perceiving speech in noise. To this end, the present study used the Wechsler Intelligence Scale for Adults (WAIS-III)^(18)^, which broadly investigates the spectrum of cognitive abilities. In this context, it is assumed that elderly people at risk of changes in cognitive abilities and hearing loss may present worse performance in speech perception skills in the presence of competitive noise. Thus, the objective of the present study was to verify the influence of verbal intellectual-cognitive skills on speech perception in noise, in the elderly with sensorineural hearing loss, considering education, age and degree of hearing loss.

## METHOD

### Ethical aspects

The observational and cross-sectional study was approved by the Research Ethics Committee of the institution of origin, under opinion number 2.390.089/2017 and CAAE 79155617.0.0000.5417. All participants signed the Free and Informed Consent Form.

### Participants

Elderly with hearing loss enrolled in the Hearing Health Service of a public-school clinic accredited by the Ministry of Health as a Hearing Health Care Service in High Complexity were selected.

Inclusion criteria:

- Mild or moderate sensorineural hearing loss, obtained from the average of thresholds at frequencies of 500 Hz, 1000 Hz, 2000 Hz and 4000 Hz^(19)^;
- Age between 60 and 89 years old, this being the standardization age limit for applying the selected cognitive test.
- If a hearing aid user (HA), time of use less than 3 months, which is the period for acclimatization to occur, and may show significant improvement in the performance of auditory skills and speech perception resulting from the new speech cues available to the HA user. Such an occurrence would be another variable to be analyzed.

Exclusion criteria:

- Asymmetric hearing losses (different degree and/or configuration of hearing loss between the right and left ears) according to the classification proposed by Hannula^(20)^;
- Changes in the middle ear detected in otorhinolaryngological medical evaluation and/or tympanometric analysis;
- Non-literate elderly people;

Thus, 36 elderly participated in the study and were subdivided into two groups. These groups were formed based on the results obtained from the Wechsler Intelligence Scale for Adults (WAIS-III) test, which assessed cognitive skills. The Gauss Curve was used with the percentage of cases under areas of the normal distribution of the intelligence quotient to classify the groups. Thus, elderly people with scores at or above average were considered to be at no risk of cognitive impairment and those with scores below average were considered to be at risk of cognitive impairment.

- Group I (GI): 24 participants without risk of cognitive impairment, with a mean age of 70.79 years, 6 female participants and 18 male participants;
- Group II (GII): 12 participants at risk of cognitive impairment, with a mean age of 70.33 years, 9 female participants and 3 male participants.

## PROCEDURES

As this is a study developed in a Hearing Health Service accredited by the Unified Health System (SUS), the population served is heterogeneous in terms of socioeconomic classification and level of education. Such data were obtained from the registration protocol of the Hearing Health Service social worker in the individual’s medical records.

### Hearing assessment

Air conduction hearing thresholds were investigated at frequencies from 0.25 to 8000 Hz, and bone conduction thresholds at frequencies from 0.5 to 4000 Hz, using an Astera/OTOMETRICS audiometer, calibrated to the ANSI-69 standard, with insert earphone (3A) and bone vibrator B-71. Tympanometry was performed using the Interacoustics model ATH 235 device, calibrated to the IEC 60645-5 standard.

### Speech perception test with competing noise

The Brazilian Hearing in Noise test (HINT-Brazil)^(21)^ consists of 12 lists of phonetically balanced sentences with twenty sentences in each list, totaling two hundred and forty available sentences. It evaluates the intelligibility of sentences, based on the Sentence Recognition Threshold (SRT/HINT-Brazil) measurement in silence and noise. The evaluation results are presented in dB/SNR (dB speech-to-noise ratio), illustrating the necessary difference between the signal presentation level and the noise presentation level, for the individual to recognize 50% of the speech material presented. In the evaluation with competitive noise (speech-weighted noise), the noise intensity is fixed and maintained at 65 dB(A), and the speech level is increased or decreased, according to the individual's successes and mistakes. The HTD (Hearing Test Device) version 7.2 microprocessor, Bio-Logic, contains the software that stores and conducts the entire test process with the recorded HINT-Brazil sentences and competing noise.

The test was applied using TDH -39 headphones, in four conditions: without competing noise, with frontal noise, with noise on the right, and with noise on the left. In the frontal noise condition: the speech signal and the competing noise are presented at the same intensity in both ears. In the noise on the right condition: the intensity of the speech signal is the same as in the contralateral ear, but the competing noise is higher in intensity in the right ear and lower in intensity in the left ear. In the noise on the left condition: the intensity of the speech signal is the same as that of the contralateral ear, but the competitive noise is presented with greater intensity in the left ear and less in the right ear.

The program provides a weighted average of noisy conditions, which is called composite noise. The sequence of sentence lists, in the different conditions, was randomly selected by the program itself to reduce variables related to attention, fatigue, participant effort and learning behavior. The answers accepted by the evaluator were: a) all words were repeated correctly; b) there was only a change in the definite and indefinite article; and c) words were added to the sentence without compromising the meaning.

The HINT-Brazil test procedure was based on two stages:

Stage 1: the first four sentences were used, which helped in the calculation for the beginning of the second stage. In Stage 1, a stimulus intensity variation of 4 dB was used. For sentence number 1, the pre-established presentation level and sentence were repeated until the individual responded correctly. With each error, the sentence was repeated at a higher intensity than the previous one. As soon as the participant responded correctly, the intensity was reduced by 4 dB, and sentence number 2 could be presented, and so on until sentence number 4, continuing the procedure of reducing and increasing intensity. After presenting the four sentences, the average of the five levels of presentation was calculated. The presentation level of sentence number 5 depended on the average presentation level of the first four sentences.

Stage 2: after Stage 1, 16 more sentences were displayed, with an intensity variation of 2 dB, that is, the speech stimulus was decreased by 2 dB if the individual answered correctly and increased by 2 dB if the answer was wrong until the list of 20 sentences was completed.

The final recognition threshold was calculated by averaging the 16 sentences from stage 2. The score was demonstrated in the ratio between speech and noise measured in decibels (dB). The more negative the value of the signal-to-noise ratio, the better the participant’s performance, since in this condition the intensity of the speech stimulus is weaker than the intensity of the noise, which demonstrates the individual’s ability to understand speech in a more difficult listening situation.

### Assessment of intellectual performance

To assess intellectual and cognitive performance, the Wechsler Intelligence Scale for Adults (WAIS-III), 3rd edition, a Brazilian adaptation and standardization instrument was used, and applied by a Psychology professional. WAIS-III is a tool that investigates the intellectual and cognitive performance of individuals from 16 to 89 years of age. It is a flexible instrument, considered the gold standard in intellectual and cognitive assessment, which allows the identification of specific cognitive domains^(18)^.

The WAIS-III structural model includes four composite indices, related to Verbal Comprehension (VC), which assesses verbal skills, such as oral comprehension; Perceptual Organization (PO), which assesses visual and spatial skills, including visual problem solving and pattern recognition; Working Memory (WM), which measures the ability to temporarily hold information in mind and manipulate it mentally; and Processing Speed (PS), which assesses how quickly the individual can process simple information and an Intellectual Intelligence Quotient (IQ). The VC consisted of the subtests Information, Similarities, and Vocabulary; the PO of Cubes and Matrix Reasoning; the WO of Digits, Arithmetic, and Sequence of Numbers and Letters; and the PS of Codes and Search Symbols.

For the present study, seven verbal subtests were selected: Vocabulary, Similarities, Arithmetic, Digits, Information, Comprehension, and Sequence of Numbers and Letters. Through this assessment, objective data on verbal comprehension skills were obtained. The results of each subtest were presented in weighted score values, verbal intelligence quotient (VIQ) classification, verbal comprehension index (VCI), and working memory index (WMI).

The data were analyzed by a psychologist according to the instrument’s regulations and classified considering the mean, minimum and maximum values and standard deviation. To classify participants, the Gauss Curve was used with the percentage of cases under areas of the normal distribution of the VIQ. The WAIS-III provides a Total Intelligence Quotient (IQ), which is a global measure of an individual’s intellectual functioning. This number represents an average score of 100, with a standard deviation of 15. A Total IQ above 100 indicates above-average performance, while a Total IQ below 100 suggests below-average performance. Individuals who presented VIQ scores between 80-110 (lower average, average and upper average) were classified as the group without risk for cognitive changes, whereas individuals who presented VIQ scores lower than 79 were members (borderline and extremely low) of the group at risk of cognitive impairment.

### Statistical analysis

The Kolmogorov-Smirnov test identified the abnormal distribution of the variables studied (age, education, speech-to-noise ratio (SNR), and WAIS-III results). In this way, non-parametric tests were used including the Mann-Whitney U Test to compare the groups and the Spearman Correlation, which verified the relationship between the variables age, degree of hearing loss, and education in the ability to perceive speech in the presence of competitive noise and in the intellectual-cognitive skills. To classify the degree of correlation, the following parameter of correlation coefficients was used: weak, when r = 0.10 to 0.30; moderate, when r = 0.40 to 0.6; strong, when r = 0.70 to 1.0. To analyze the results, the SPSS software version 17 was used and a significance level of p≤0.05 was adopted.

## RESULTS

**Figure 1:**
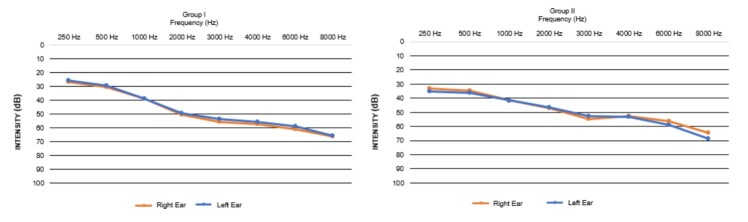
Average hearing thresholds in the right and left ears, for groups I and II

In Figure 1 it is possible to view the descriptive results of the average hearing thresholds in the right and left ears, as well as the audiometric configuration in both groups, which presented similar auditory profiles.

Table 1 shows the first quartile, the median, and the third quartile of the results found in HINT-Brazil in the binaural modality (dB/SNR), and the p-value obtained by the Mann-Whitney U Test in the comparison between the groups. The results showed a significant difference only in the noise on the left condition.

**Table 1:** First quartile, median and third quartile of the HINT-Brazil result in the binaural modality and p value obtained by the Mann-Whitney U Test when comparing the groups

**HINT- Binaural modality**	**1Q**		**Median**		**3Q**		**p**
	**GI**	**GII**	**GI**	**GII**	**GI**	**GII**	
HINT – no noise (dB)	46.80	47.40	53.30	50.65	59.27	55.07	0.40
HINT - frontal noise (dB)	-0.65	-0.97	0.40	-0.40	1.62	0.05	0.10
HINT – right noise (dB)	-4.62	-4.70	-1.30	-2.55	2.02	0.07	0.50
HINT – left noise (dB)	-4.07	-5.57	-2.00	-4.50	0.37	-2.67	0.00^*^
HINT – composite noise (dB)	-2.30	–3.07	-0.40	-1.65	1.25	-0.07	0.10

* Statistically significant; p≤0,05

Caption: HINT-Brazil = Hearing in Noise Test; dB = decibel; GI = no risk of cognitive impairment; GII = at risk of cognitive impairment; 1Q = first quartile; 3Q = third quartile

The classification of level of educational for GI and GII is shown in Table 2.

**Table 2:** Education level and p value when comparing groups using the Mann-Whitney U Test

	**GI (n=24)**		**GII (n=12)**		**Mann-Whitney U Test**
Education	N	%	n	%	p
Higher	7	29.2	0	0	U=68.5 p= 0.0^*^
Middle School Complete	4	16.7	0	0	
Middle School incomplete	6	25.0	0	0	
Elementary School complete	0	0	2	16.7	
Elementary School incomplete	7	29.2	3	25	
Literate	0	0	7	58.3	
Not information	1	4.2	0	0	

* Statistically significant; p≤0,05

Caption: n = number of participants; GI = no risk of cognitive impairment; GII = at risk of cognitive impairment

The impact of age, degree of hearing loss, and level of education in the results obtained in the WAIS-III and HINT-Brazil tests were analyzed using the Spearman correlation test shown in Table 3.

**Table 3:** Correlations between the variables age, degree of hearing loss, education level and the tests WAIS-III and HINT-Brazil

Variables	Age		Degree RE		Degree LE		Education level	
	GI	GII	GI	GII	GI	GII	GI	GII
HINT								
Frontal noise	0.167	0.620^*^	0.466*	0.745**	0.554**	0.745**	-0.323	-0.197
Right noise	0.199	0.056	0.492*	0.512	0.560**	0.512	-0.311	-0.000
Left noise	0.048	0.107	0.614^**^	0.436	0.666**	0.436	-0.514*	-0.170
Composite noise	0.141	0.474	0.658**	0.717**	0.709**	0.717**	-0.368	-0.315
**Variables**	**Age**		**Degree RE**		**Degree LE**		**Education level**	
	**GI**	**GII**	**GI**	**GII**	**GI**	**GII**	**GI**	**GII**
**WAIS-III**								
Vocabulary	0.449*	-0.057	-0.032	-0.400	0.019	-0.400	0.171	0.535
Similarity	0.355	0.341	-0.085	0.131	-0.038	0.131	0.193	0.674*
Arithmetic	0,424*	-0.022	-0.202	0.507	-0.171	0.507	0.096	0.064
Digits	0.074	-0.089	-0.280	0.346	-0.254	0.346	0.078	0.008
Information	0.272	-0.240	-0.278	0.405	-0.346	0.405	0.697**	0.441
Comprehension	0.482*	0.024	0.019	-0.258	-0.019	-0.258	0.111	0.225
Sequence of letters and numbers	0.134	-0.329	-0.187	-0.159	-0.188	-0.159	0.090	0.215
VIQ	0.430*	-0.081	-0.243	0.129	-0.231	0.129	0.407*	0.169
VCI	0.400	0.207	-0.154	-0.079	-0.162	-0.079	0.427*	0.711**
WMI	0.274	-0.247	-0.281	-0.259	-0.280	-0.259	0.175	0.010

Spearman correlation

* Significant for p≤0.05;

** Significant for p≤0.01

Caption: RE = right ear; LE = left ear; GI = no risk of cognitive impairment; GII = at risk of cognitive impairment; VIQ = verbal intelligence quotient; VCI = verbal comprehension index; WMI = working memory index; HINT-Brazil = Hearing in Noise Test; WAIS-III = Wechsler Intelligence Scale for Adults

## DISCUSSION

One of the objectives of the study was to verify the influence of verbal intellectual-cognitive skills on the perception of speech in competitive noise in the elderly with sensorineural hearing loss. Thus, a difference was observed between the groups with and without risk of cognitive changes in the ability to perceive speech in the condition noise on the left (Table 1), with elderly from GII (at risk) performing better when compared to the elderly from GI (no risk). Therefore, considering the groups studied, speech perception in the presence of competitive noise was not influenced by cognitive changes.

The absence of a relationship between changes in cognitive function and worse performance in speech-in-noise tests has already been reported in previous studies, regardless of the presence or absence of hearing loss^(22,23)^.

On the other hand, older adults without hearing loss, with good temporal auditory processing skills, and with reduced working memory capacity and processing speed showed worse speech perception performance in noise when compared to younger adults. The authors did not attribute this finding solely to age-related decline in cognitive aspects^(24)^ but highlighted interindividual variability and hearing status. Given this, it is clear that the impairment in the ability to perceive speech in noise is impacted by other factors, especially when the population is elderly.

This contradiction is also evident in literature review studies. A total of 20 studies were analyzed and a link was found between speech perception in competitive noise and cognition in different individuals, without and with hearing loss (HA users). However, no cognitive test showed a significant result considering all the variables studied. Thus, the results of this review highlighted inconsistencies between the cognitive skills assessed and the speech perception tests in competitive noise. For example, an association was only found between working memory performance and intelligence quotient (IQ) in speech tests with competitive noise^(25)^.

In a review with meta-analysis, no significant difference was observed between the working memory of young adults in the sentence test in the presence of competitive noise, in 16 studies evaluated. It was concluded that working memory makes little contribution to individual differences in speech perception in competitive noise among young listeners. However, it was observed that the studies included in the review had a small sample and a wide confidence interval^(15)^.

In the systematic review with meta-analysis, Dryden et al.^(8)^ analyzed 25 studies. They found a weak general association (r = 0.31) between cognitive performance (attention, memory, executive function, IQ, and processing speed) and the ability to perceive speech in noise in hearing adults with mild to moderate hearing loss. They also observed heterogeneity between study methodologies, and great variability in this association depending on cognitive function, speech perception test, and masking noise assessed^(8)^.

Specifically to the HINT-Brazil results in the binaural presentation, it was observed that the elderly population at risk of cognitive impairment performed better in the noise on the left condition (Table 1). Given this result, it is important to understand the functional mechanisms that occur in each HINT-Brazil listening condition.

The HINT-Brazil condition with noise on the left is more favorable listening when compared to the HINT-Brazil with frontal noise condition and noise on the right condition^(6)^. When analyzing each of them comparatively, it is observed that, in the frontal noise condition, listening is more unfavorable, as the noise is presented at the same intensity as the speech signal bilaterally. There are reports that in the most challenging signal condition, the individual uses superior cognitive skills such as working memory, attention control and processing speed in performing speech perception in noise (top-down processing)^(6)^. However, in the present study, individuals at risk of cognitive impairment (GII) did not show worse performance in the test compared to GI (Table 1).

In the HINT-Brazil condition on the right, the noise is presented more intensely in the right ear (unshadowed noise) and weaker in the left ear (head-shadowed noise), while the sentences are presented at the same intensity in both ears. For the elderly, this condition can also represent a considerable degree of difficulty, since, despite the S/N ratio being more positive in relation to the right ear, there is a disadvantage in speech perception in the left ear with increasing age^(26)^. With aging, morphological changes are observed in the cerebral cortices, with greater deterioration of the right hemisphere, in addition to degeneration of the corpus callosum, the structure of the nervous system responsible for transferring information between one hemisphere and another, which causes a decline in interhemispheric transmission between the ears with increasing age^(26,27)^. In the present study, performance between groups was similar in this test condition, that is, there was no significant impact of cognitive decline on the performance of individuals in group GII.

In the HINT-Brazil condition on the left, the speech signal is presented at the same intensity in the contralateral ear (right), but with stronger competing noise intensity in the left ear (unshadowed noise) and weaker noise on the right (head-shadowed). Therefore, speech perception is more favorable since the SRN is greater in the right ear. In the present study, for elderly people at risk of cognitive impairment, this condition was the least challenging in the test and, therefore, performance was better. The right ear advantage, with or without competing noise, is expected in normal speech processing, as reported in studies^(26,27)^. However, the result found in the present study emphasizes that even in the face of cognitive decline, attention in the rehabilitation process regarding the perception of speech in noise, especially with the adaptation of the hearing aid, should continue to focus on the right ear due to its advantage, if binaural adaptation of the hearing aid is not feasible^(27)^.

From the results shown in Table 2, it is possible to verify that there was a difference between the groups in relation to education, which reveals possible interference of this condition in the perception of speech in noise, since the performance in HINT-Brazil was different between the groups (Table 1). GII (at risk of cognitive changes) had worse levels of education. However, in this study, the level of education was not related to the HINT-Brazil test in the group of elderly people at risk of cognitive changes. In the group without risk of cognitive changes, there was a moderate positive correlation in the noise on the left condition of the HINT-Brazil. Thus, it was observed that the higher the educational level of the elderly without changes in cognitive function, the lower the SNR. According to a previous study, for a one-year increase in education, the SNR decreases by 0.40 dB, on average^(5)^.

It is noteworthy that, although speech perception in noise is the dependent variable in this study, there is a relationship between educational factors and cognitive status^(16,17)^, that is, cognition is dependent on this qualitative variable. Thus, it is understood that when grouping based on the results of the WAIS-III test, there was the expectation that the group without risk for cognitive changes would contain elderly people with a higher level of education. The data presented in Table 2 confirm this evidence.

Regarding the variable age, there was a moderate correlation in GII (risk of cognitive alteration) only in the HINT-Brazil frontal noise condition, that is, the older the age, the worse the performance in this condition (Table 3). Increasing age is an aggravating factor in speech perception in competitive noise^(6,25)^. However, the variable age did not interfere with the other HINT-Brazil conditions. Given this result, it is noteworthy that depending on the condition of the speech test in noise, that is, the laterality of presentation of speech in competitive noise, the impairment may not be evident with advancing age. Furthermore, it is considered that other factors, in addition to increasing age, may interfere with the perception of speech in competitive noise, such as inter-individual variability^(24)^.

There was a weak correlation between the variable age and the performance of some WAIS-III tests, such as vocabulary, arithmetic, comprehension, and verbal intelligence quotient for the elderly in the GI. This finding shows that elderly people without risk of cognitive alteration may present worse results in specific cognitive tests influenced by advancing age^(7)^, but still with preserved cognitive skills that differentiate them regarding cognitive status. On the other hand, absolute ages did not influence the result in any specific test for the elderly in GII. Given these findings related to the study groups, it can be seen that when cognitive impairment is present, age is not the dependent variable, that is, although there is a cognitive decline with advancing age, this may occur due to other factors, such as life experience, physical activity, education level, socioeconomic factors, sleep quality, sensory deprivation, and healthy eating habits^(16,17)^.

It is important to highlight that all participants had hearing loss (Figure 1), and when classifying the elderly into groups I and II based on the WAIS-III results, a balanced distribution was observed regarding the degree of hearing loss between the groups, i.e., 33.3% of individuals with mild sensorineural hearing loss and 66.7% with moderate sensorineural hearing loss in each group. In both groups there was a highly significant positive correlation between the degree of hearing loss and the perception of speech in noise (Table 3), that is, the greater the degree of hearing loss, the greater the S/N ratio, demonstrating worse performance in speech perception when the noise was louder, as already reported in the literature^(14)^.

Thus, regardless of whether or not there is a risk of cognitive function deficits, the greater the degree of hearing loss in the elderly, the greater the difficulty in difficult listening environments (Table 3). There is a gap in knowledge about the relationship between the degree of hearing loss and cognition, reported in recent studies^(12,13,28,29)^. In the present study, the degree of mild or moderate hearing loss was not decisive for classifying participants into GI or GII.

In this study, the ability to perceive speech in the presence of competitive noise was influenced by the degree of hearing loss and level of education. However, it was not possible to confirm the interference of cognitive resources in the HINT-Brazil test. Therefore, the study hypothesis was not confirmed.

Finally, given all the considerations reported in the present study and the scientific evidence highlighted in the literature, there is a lack of consistent evidence to understand whether the difficulties in performing speech perception in noise are due to the age-related deterioration of cognitive functions in a specific domain, such as working memory^(25)^ due to age-related hearing loss^(11,30)^ and/or other aging-related impairment^(12,23,24)^.

Regarding the limitation of the study, statistical methods appropriate to the sample size were used, enabling a better understanding of the uncertainty and variability of the data, and confirming that the results found are valid. However, there were a small number of elderly at risk of cognitive changes, which may have been a weakness in the research.

## CONCLUSION

The decline in verbal cognitive skills did not affect speech perception in noise in the elderly with hearing loss. The speech perception ability was influenced by the variables degree of hearing loss and level of education in both elderly people at risk of cognitive impairment and those without risk. Finally, educational level interfered with the performance of cognitive function.
