# Supplementary material for: Elderly people with hearing loss and cognitive decline: speech perception performance in noise
Source: Codas. 2024 Jun 14;36(3):e20230094. doi: 10.1590/2317-1782/20242023094en (PMC466999; doi:10.1590/2317-1782/20242023094en)
Supplement: Translation

# Translation: Idosos com perda auditiva e declínio cognitivo: desempenho da percepção de fala no ruído

**DOI:** 10.1590/2317-1782/20242023094pt

**Authors:** Maria Julia Ferreira Cardoso, Kátia de Freitas Alvarenga, Maria de Lourdes Merighi Tabaquim, Tatiana de Andrade Lopes, Orozimbo Alves Costa, Lilian Cássia Bórnia Jacob

**Affiliations:** 1 Programa de Pós-graduação em Fonoaudiologia, Faculdade de Odontologia de Bauru – FOB, Universidade de São Paulo – USP - Bauru (SP), Brasil.; 2 Departamento de Fonoaudiologia, Faculdade de Odontologia de Bauru – FOB, Universidade de São Paulo – USP - Bauru (SP), Brasil.

**Keywords:** Idoso, Perda Auditiva, Percepção da Fala, Cognição, Declínio Cognitivo

## Abstract

**Objetivo:**

Verificar a influência das habilidades intelectuais-cognitivas verbais na percepção de fala no ruído, em idosos com perda auditiva sensorioneural, considerando a escolaridade, a idade e o grau da perda auditiva.

**Método:**

Participaram 36 idosos entre 60 e 89 anos com perda auditiva sensorioneural bilateral, que após avaliação psicológica por meio do Wechsler Intelligence Scale for Adults (WAIS III), foram divididos em (GI) 24 idosos sem alteração cognitiva e (GII) 12 idosos com risco de alteração cognitiva. Foram submetidos à avaliação otorrinolaringológica, entrevista audiológica, audiometria tonal liminar e a avaliação da percepção de fala no ruído por meio do Hearing in Noise Test (HINT-Brasil). O teste estatístico U de Mann-Whitney comparou os resultados entre os grupos, e a correlação de Spearman verificou as variáveis idade, grau da perda auditiva e nível de escolaridade.

**Resultados:**

Não houve diferença entre os grupos na habilidade de percepção de fala no ruído, exceto na condição ruído à esquerda, no qual o GII apresentou melhor desempenho no HINT-Brasil. O grau da perda auditiva e o nível de escolaridade influenciaram na percepção de fala no ruído. O nível de escolaridade teve correlação com os resultados do WAIS III.

**Conclusão:**

O declínio das habilidades intelectuais-cognitivas verbais não interferiu na percepção de fala no ruído nos idosos com perda auditiva. O grau da perda auditiva e o nível de escolaridade influenciaram no desempenho dos idosos no teste de percepção de fala no ruído. O desempenho nas habilidades cognitivas verbais variou com o nível de escolaridade.

## INTRODUÇÃO

A perda auditiva relacionada à idade, é uma das condições crônicas de saúde mais prevalente na terceira idade resultante dos efeitos cumulativos do envelhecimento no sistema auditivo^(1)^. As características da perda auditiva relacionada à idade são ser progressiva, do tipo sensorioneural bilateral, simétrica e com configuração audiométrica descendente com acometimento maior nas altas frequências^(2)^.

Com o aumento da expectativa de vida no Brasil^(3)^, os serviços de saúde auditiva recebem cada vez mais idosos com queixa em relação à audibilidade e de compreensão de fala em ambientes desfavoráveis. O aumento da demanda está relacionado à busca pela aquisição do aparelho auditivo, visando amenizar as dificuldades auditivas e melhorar a queixa principal, que se caracteriza pela dificuldade de percepção de fala, especialmente na presença de ruído competitivo^(4,5)^.

Esta dificuldade não é justificada somente pelas alterações no sistema auditivo periférico, mas também por alterações no sistema nervoso auditivo central^(6,7)^. Paralelamente ao processo de alterações auditivas relacionadas ao envelhecimento, poderá manifestar-se o declínio nas habilidades intelectuais e nas funções cognitivas, que vem sendo associado a maiores alterações na percepção de fala no ruído competitivo^(5)^, ou seja, há evidências de associação entre pior desempenho no teste de percepção de fala no ruído competitivo e o declínio das funções cognitivas^(8)^.

Contudo, há diferentes hipóteses sobre os mecanismos etiológicos envolvidos com perda auditiva relacionada à idade e declínio das funções cognitivas^(1)^: hipótese de carga cognitiva: é teorizada que a perda auditiva leva à deterioração dos sinais auditivos, sendo recrutados maiores recursos cognitivos para o processamento perceptual auditivo e desvio de outros recursos cognitivos para um escuta com esforço auditivo, ocasionando um esgotamento da reserva cognitiva^(2)^; hipótese de causa comum: afirma que tanto a perda auditiva quanto às alterações das funções cognitivas são resultantes de um processo neurodegenerativo comum no cérebro envelhecido^(3)^; hipótese de cascata: explica que a perda auditiva periférica afeta a estrutura cerebral diretamente por meio de informações sensoriais degradadas, ou seja, a perda auditiva relacionada à idade está associada a menor volume cerebral e a taxas aceleradas de atrofia cerebral; e^(4)^ sobrediagnóstico ou hipótese precursora: a perda auditiva impacta o desempenho em certos testes neuropsicológicos, ou seja, como as instruções verbais ou tarefas que dependem consideravelmente da audição são utilizadas durante os testes cognitivos, os indivíduos com perda auditiva podem ficar em desvantagem^(9,10)^. Diante dessa realidade multifacetada, configura-se a complexa relação entre envelhecimento, perda auditiva, cognição e percepção de fala no ruído.

Independente da teoria que embasa tal relação, a perda auditiva foi considerada como fator de risco modificável para alteração da função cognitiva e suas implicações, representando a maior proporção de ameaça atribuível em comparação a outras variáveis analisadas como diabetes, obesidade, hipertensão, dentre outras^(11,12)^.

Por outro lado, existem achados controversos na literatura, uma vez que estudos não encontraram relação entre grau de perda auditiva e cognição^(12-14)^ e não observaram relação entre cognição e habilidade de percepção de fala no ruído^(15)^. Porém, a metanálise de uma revisão sistemática apontou que as funções cognitivas de controle inibitório, memória de trabalho, memória episódica e velocidade de processamento são importantes para a habilidade de percepção de fala no ruído^(8)^. Portanto, são temáticas atuais no estudo da perda auditiva relacionada ao envelhecimento.

Outra variável considerada importante nos estudos que envolvem a cognição é a escolaridade^(16)^, pois até em indivíduos que não apresentam alteração da função cognitiva, quanto menor o nível de escolaridade, menor a pontuação nos testes de função cognitiva, portanto, a sua influência no status cognitivo^(16,17)^ deve ser considerada em estudos que envolvam a avaliação de habilidades cognitivas.

Há escassez de estudos na literatura nacional que investigaram a associação entre perda auditiva relacionada à idade, as alterações das funções cognitivas e a dificuldade de percepção de fala no ruído. Para tal, o presente estudo utilizou o Wechsler Intelligence Scale for Adults (WAIS-III)^(18)^ que investiga de forma ampla o espectro de capacidades cognitivas. Nesse contexto, têm-se como pressuposto que idosos com risco de alterações nas habilidades cognitivas e perda auditiva podem apresentar pior performance na habilidade de percepção de fala na presença de ruído competitivo. Assim, o objetivo do presente estudo foi verificar a influência das habilidades intelectuais-cognitivas verbais na percepção de fala no ruído, em idosos com perda auditiva sensorioneural, considerando a escolaridade, a idade e o grau da perda auditiva.

## MÉTODO

### Aspectos éticos

O estudo, do tipo observacional e transversal, foi aprovado pelo Comitê de Ética em Pesquisa da instituição de origem, sob o parecer 2.390.089/2017 e CAAE 79155617.0.0000.5417. Todos os participantes assinaram o Termo de Consentimento Livre e Esclarecido.

### Participantes

Foram selecionados os idosos com perda auditiva matriculados no Serviço de Saúde Auditiva de uma clínica escola pública, credenciado no Ministério da Saúde como Serviço de Atenção à Saúde Auditiva na Alta Complexidade.

Critérios de inclusão:

- Perda auditiva sensorioneural de grau leve ou moderado, obtidos a partir da média dos limiares nas frequências de 500 Hz, 1000 Hz, 2000 Hz e 4000 Hz^(19)^;
- Idade entre 60 e 89 anos, sendo esta a idade limite de padronização para aplicação do teste cognitivo selecionado.
- Se usuário de aparelho de amplificação sonora individual (AASI), o tempo de uso foi inferior a 3 meses, período considerado para a ocorrência da aclimatização, podendo apresentar uma melhora significativa nos desempenhos das habilidades auditivas e de percepção de fala decorrente das novas pistas de fala disponíveis ao usuário da amplificação. Tal ocorrência seria outra variável a ser analisada.

Critérios de exclusão:

- Perdas auditivas assimétricas (grau e/ou configuração da perda auditiva diferentes entre as orelhas direita e esquerda de acordo com classificação proposta por Hannula^(20)^;
- Alterações na orelha média detectadas na avaliação médica otorrinolaringológica e/ou na análise timpanométrica;
- Idosos não alfabetizados;

Desta forma, participaram do estudo 36 idosos que foram subdivididos em dois grupos. Tais grupos foram formados a partir dos resultados obtidos no teste Wechsler Intelligence Scale for Adults (WAIS-III) que avaliou as habilidades cognitivas. Utilizou-se a Curva de Gauss com a porcentagem de casos sob áreas da distribuição de normalidade do quociente de inteligência para classificação dos grupos. Assim, os idosos que apresentaram escores na média ou superior da média foram considerados sem risco de alteração cognitiva e aqueles que apresentaram escores inferiores da média com risco de alteração cognitiva.

- Grupo I (GI): 24 participantes sem risco de alteração cognitiva, com média de idade de 70,79 anos, sendo 6 do sexo feminino e 18 do masculino;
- Grupo II (GII): 12 participantes com risco de alteração cognitiva, com média de idade de 70,33 anos, 9 do sexo feminino e 3 do masculino.

## PROCEDIMENTOS

Por se tratar de um estudo desenvolvido em um Serviço de Saúde Auditiva credenciado ao Sistema Único de Saúde (SUS), a população atendida é heterogênea quanto à classificação socioeconômica e nível de escolaridade Tais dados foram obtidos a partir do protocolo de registro da assistente social do Serviço de Saúde Auditiva no prontuário do indivíduo.

### Avaliação audiológica

Os limiares auditivos por condução aérea foram pesquisados nas frequências de 0,25 a 8000 Hz, e os limiares por condução óssea nas frequências de 0,5 a 4000 Hz, por meio do audiômetro da marca Astera/OTOMETRICS, calibrado no padrão ANSI-69, com fones de inserção (3A) e vibrador B-71. A timpanometria foi realizada por meio do imitanciômetro modelo ATH 235 da Interacoustics, calibrado no padrão IEC 60645-5.

### Teste de percepção de fala em situação com ruído competidor

O teste Hearing in Noise brasileiro (HINT-Brasil)^(21)^ é constituído por 12 listas de sentenças foneticamente balanceadas com vinte sentenças em cada lista, totalizando duzentos e quarenta sentenças disponíveis. Avalia a inteligibilidade de sentenças, a partir da medida do Limiar de Reconhecimento de Sentenças (LRF/HINT-Brasil) no silêncio e no ruído. Os resultados da avaliação são apresentados em dB/FR (dB relação fala-ruído), ilustrando a diferença necessária entre o nível de apresentação do sinal e o nível de apresentação do ruído, para que o indivíduo reconheça 50% do material de fala apresentado. Na avaliação com o ruído competitivo (*speech-weighted noise)*, a intensidade de ruído é fixa e mantida em 65 dB(A) e o nível da fala é aumentado ou diminuído, de acordo com os acertos e erros do indivíduo. O microprocessador HTD (Hearing Test Device) versão 7.2, *Bio-Logic*, contém o software que armazena e conduz todo o processo do teste com as sentenças gravadas do HINT-Brasil e com o ruído competidor.

O teste foi aplicado por meio de fones TDH -39, em quatro condições: sem ruído competitivo, com ruído frontal, com ruído à direita e com ruído à esquerda. Na condição ruído frontal: o sinal de fala e o ruído competitivo é apresentado na mesma intensidade em ambas as orelhas. Na condição ruído à direita: a intensidade do sinal de fala é a mesma da orelha contralateral, mas o ruído competitivo em maior intensidade na orelha direita e menor intensidade na orelha esquerda. Na última condição, o ruído à esquerda: a intensidade do sinal de fala é a mesma da orelha contralateral, mas o ruído competitivo apresentado com maior intensidade na orelha esquerda e menor na orelha direita.

O programa fornece uma média ponderada das condições com ruído, a qual denomina ruído composto. A sequência das listas de sentenças, nas diferentes condições, foi selecionada de forma aleatória pelo próprio programa a fim de reduzir variáveis relacionadas à atenção, ao cansaço, esforço do participante e ao comportamento de aprendizagem. As respostas aceitas pelo avaliador foram: a) todas as palavras foram repetidas corretamente; b) houve apenas mudança do artigo definido e indefinido; e c) foram adicionadas palavras à sentença sem comprometer o sentido.

O procedimento do teste HINT-Brasil baseou-se em duas etapas:

Primeira etapa: foram usadas as quatro primeiras sentenças, que auxiliaram no cálculo para o início da segunda etapa. Na etapa 1 foi utilizada a variação de estímulo de intensidade de 4 dB. Para a sentença número 1, o nível de apresentação preestabelecido e a sentença foram repetidos até que o indivíduo respondesse corretamente. A cada erro, a sentença foi repetida em uma intensidade acima da anterior. Assim que o participante respondia corretamente, a intensidade era reduzida em 4 dB, podendo ser apresentada a sentença de número 2, e assim por diante até a sentença de número 4, dando continuidade no procedimento de redução e aumento da intensidade. Após a apresentação das quatro sentenças, foi realizado o cálculo de média dos cinco níveis de apresentação. O nível de apresentação da sentença de número 5 dependeu da média do nível de apresentação das quatro primeiras sentenças.

Na etapa 2: após a primeira etapa, foram exibidas mais 16 sentenças, com variação da intensidade de 2 dB, ou seja, o estímulo de fala foi diminuído em 2 dB se o indivíduo respondesse certo e aumentado em 2 dB caso a resposta apresentasse errada, até que se finalizasse a lista de 20 sentenças.

O limiar de reconhecimento final foi calculado pela média das 16 sentenças da fase 2. A pontuação foi demonstrada na relação entre a fala e o ruído medida em decibel (dB). Quanto mais negativo o valor da relação sinal-ruído, melhor o desempenho do participante, uma vez que, nesta condição a intensidade do estímulo de fala está mais fraca que a intensidade do ruído, o que demonstra a habilidade do indivíduo em compreender a fala em uma situação de escuta mais difícil.

### Avaliação do desempenho intelectual

Para a avaliação do desempenho intelectual e cognitivo, utilizou-se o instrumento Wechsler Intelligence Scale for Adults (WAIS-III), 3ª edição, adaptação e normatização brasileira aplicado por um profissional da Psicologia. O WAIS-III é uma ferramenta que investiga o desempenho intelectual e cognitivo, para indivíduos a partir de 16 anos de idade até 89 anos. É um instrumento flexível considerado padrão-ouro para a avaliação intelectual e cognitiva que permite a identificação de domínios cognitivos específicos^(18)^.

O modelo estrutural do WAIS-III contempla quatro índices compostos, relacionados à Compreensão Verbal (CV) que avalia habilidades verbais, como compreensão oral; Organização Perceptual (OP) que avalia habilidades visuais e espaciais, incluindo resolução de problemas visuais e reconhecimento de padrões; Memória Operacional (MO) que mede a capacidade de manter informações em mente temporariamente e manipulá-las mentalmente; e Velocidade de Processamento (VP) que avalia a rapidez com que o indivíduo pode processar informações simples e um Quociente Intelectual de Inteligência (QI). O CV constituiu-se pelos subtestes Informação, Semelhanças e Vocabulário; a OP por Cubos, Raciocínio Matricial e Cubos e a MO por Dígitos, Aritmética e Sequência de Números e Letras; e a VP, com os subtestes Códigos e Procurar Símbolos.

Para o presente estudo, foram selecionados sete subtestes verbais: Vocabulário, Semelhanças, Aritmética, Dígitos, Informação, Compreensão e Sequência de Números e Letras. Por meio dessa avaliação, foram obtidos dados objetivos sobre habilidade de compreensão verbal. Os resultados de cada subteste foram apresentados em valores de escores ponderados, classificação do quociente de inteligência verbal (QIV), índice de compreensão verbal (ICV) e índice de memória operacional (IMO).

Os dados foram analisados por uma psicóloga de acordo com as normativas do instrumento e classificados considerando a média, valores mínimos e máximos e desvio padrão. Para a classificação dos participantes utilizou-se a Curva de Gauss com a porcentagem de casos sob áreas da distribuição de normalidade do QIV. O WAIS-III fornece um Quociente Inteligência (QI) Total que é uma medida global do funcionamento intelectual do indivíduo. Este número representa uma pontuação média de 100, com um desvio padrão de 15. Um QI Total acima de 100 indica um desempenho acima da média, enquanto um QI Total abaixo de 100 sugere um desempenho abaixo da média. Os indivíduos que apresentaram escores do QIV entre 80-110 (médio inferior, média e média superior) foram classificados como o grupo sem risco para alteração cognitiva, já os indivíduos que apresentaram escores do QIV inferiores 79 foram integrantes (limítrofe e extremamente baixo) do grupo com risco de alteração cognitiva.

### Análise estatística

O teste Kolmogorov-Smirnov identificou a distribuição anormal das variáveis estudadas (idade, escolaridade, relação fala-ruído (F/R), e resultados do WAIS-III). Desta forma, foram utilizados testes não paramétricos constituídos pelo Teste U de Mann-Whitney para a comparação entre os grupos e Correlação Spearman, na qual verificou a relação das variáveis idade, grau da perda auditiva e escolaridade na habilidade de percepção de fala na presença de ruído competitivo e nas habilidades intelectuais-cognitivas. Como classificação do grau de correlação, utilizou-se o seguinte parâmetro dos coeficientes de correlação: fraco, quando r = 0,10 até 0,30; moderado, r = 0,40 até 0,6; forte, quando r = 0,70 até 1,0. Para análise dos resultados, foi utilizado o software SPSS versão 17 e adotado o nível de significância de p≤0,05.

## RESULTADOS

Na Figura 1 é possível visualizar os resultados descritivos da média dos limiares auditivos nas orelhas direita e esquerda, bem como a configuração audiométrica, em ambos os grupos, que apresentaram perfil auditivo semelhantes.

**Figura 1:** Média dos limiares auditivos nas orelhas direita e esquerda, para os grupos I e II

Na Tabela 1 pode ser visualizada o primeiro quartil, a mediana e o terceiro quartil dos resultados encontrados no HINT-Brasil na modalidade binaural (dB/FR), e o valor de p obtido pelo Teste U de Mann-Whitney na comparação entre os grupos. Os resultados apontaram diferença significativa somente na condição ruído à esquerda.

**Tabela 1:** Primeiro quartil, mediana e terceiro quartil do resultado do HINT-Brasil na modalidade binaural e valor de p obtido pelo Teste U de Mann-Whitney na comparação entre os grupos

**HINT-Modalidade Binaural**	**1Q**		**Mediana**		**3Q**		**P**
	**GI**	**GII**	**GI**	**GII**	**GI**	**GII**	
HINT - sem ruído (dB)	46,80	47,40	53,30	50,65	59,27	55,07	0,40
HINT - ruído frontal (dB)	-0,65	-0,97	0,40	-0,40	1,62	0,05	0,10
HINT - ruído à direita (dB)	-4,62	-4,70	-1,30	-2,55	2,02	0,07	0,50
HINT - ruído à esquerda (dB)	-4,07	-5,57	-2,00	-4,50	0,37	-2,67	0,00^*^
HINT - ruído composto (dB)	-2,30	–3,07	-0,40	-1,65	1,25	-0,07	0,10

* Estatisticamente significante; p≤0,05

Legenda: HINT-Brasil = Hearing in Noise Test; dB = decibel; GI = sem risco de alteração cognitivo; GII = com risco de alteração cognitiva; 1Q = primeiro quartil; 3Q = terceiro quartil

A classificação do nível de escolaridade para os grupos GI e GII está apresentada na Tabela 2.

**Tabela 2:** Nível de escolaridade e valor de p na comparação entre os grupos por meio do Teste U de Mann-Whitney

	**GI (n=24)**		**GII (n=12)**		**Teste U de Mann-Whitney**
Escolaridade	n	%	n	%	P
Superior	7	29,2	0	0	U=68,5 p= 0,0^*^
Fund. Ciclo II completo	4	16,7	0	0	
Fund. Ciclo II Incompleto	6	25,0	0	0	
Fund. Ciclo I completo	0	0	2	16,7	
Fund. Ciclo I incompleto	7	29,2	3	25	
Alfabetizado	0	0	7	58,3	
Sem informação	1	4,2	0	0	

* Estatisticamente significante; p≤0,05

Legenda: n = número de participantes; GI = sem risco de alteração cognitivo; GII = com risco de alteração cognitiva

A interferência da idade, do grau da perda auditiva e do nível de escolaridade nos resultados obtidos nos testes WAIS-III e HINT-Brasil, analisada por meio do teste de correlação de Spearman, pode ser observada na Tabela 3.

**Tabela 3:** Correlações entres as variáveis idade, grau da perda auditiva, nível de escolaridade e os testes WAIS-III e HINT-Brasil

Variáveis	Idade		Grau OD		Grau OE		Nível de escolaridade	
	GI	GII	GI	GII	GI	GII	GI	GII
HINT								
Ruído frontal	0,167	0,620*	0,466*	0,745**	0,554**	0,745**	-0,323	-0,197
Ruído à direita	0,199	0,056	0,492*	0,512	0,560**	0,512	-0,311	-0,000
Ruído à esquerda	0,048	0,107	0,614^**^	0,436	0,666**	0,436	-0,514*	-0,170
Ruído composto	0,141	0,474	0,658**	0,717**	0,709**	0,717**	-0,368	-0,315
**Variáveis**	**Idade**		**Grau OD**		**Grau OE**		**Nível de escolaridade**	
	**GI**	**GII**	**GI**	**GII**	**GI**	**GII**	**GI**	**GII**
**WAIS-III**								
Vocabulário	0,449*	-0,057	-0,032	-0,400	0,019	-0,400	0,171	0,535
Semelhança	0,355	0,341	-0,085	0,131	-0,038	0,131	0,193	0,674*
Aritmética	0,424*	-0,022	-0,202	0,507	-0,171	0,507	0,096	0,064
Dígitos	0,074	-0,089	-0,280	0,346	-0,254	0,346	0,078	0,008
Informação	0,272	-0,240	-0,278	0,405	-0,346	0,405	0,697**	0,441
Compreensão	0,482^*^	0,024	0,019	-0,258	-0,019	-0,258	0,111	0,225
Seq. de letras e nº	0,134	-0,329	-0,187	-0,159	-0,188	-0,159	0,090	0,215
QIV	0,430*	-0,081	-0,243	0,129	-0,231	0,129	0,407*	0,169
ICV	0,400	0,207	-0,154	-0,079	-0,162	-0,079	0,427*	0,711**
IMO	0,274	-0,247	-0,281	-0,259	-0,280	-0,259	0,175	0,010

Correlação de Spearman

* Significativo para p≤0,05;

** Significativo para p≤0,01

Legenda: OD = orelha direita; OE = orelha esquerda; GI = sem risco de alteração cognitiva; GII = com risco de alteração cognitiva; QIV = quociente de inteligência verbal; ICV = índice de compreensão verbal; IMO = índice de memória operacional; HINT-Brasil = Hearing in Noise Test; WAIS-III = Wechsler Intelligence Scale for Adults

## DISCUSSÃO

Um dos objetivos do estudo foi verificar a influência das habilidades intelectual-cognitivas verbais na percepção de fala com ruído competitivo, em idosos com perda auditiva sensorioneural. Assim, observou-se diferença entre os grupos com e sem risco de alteração cognitiva na habilidade de percepção de fala na condição de ruído à esquerda (Tabela 1), sendo que os idosos do GII (com risco) apresentaram desempenho melhor quando comparado aos idosos do GI (sem risco). Desta forma, considerando os grupos estudados, a percepção de fala na presença de ruído competitivo não sofreu influência da alteração cognitiva.

A ausência de relação entre a alteração da função cognitiva e o pior desempenho nos testes de fala no ruído já foi reportada em estudos prévios, independente da presença ou não da perda auditiva^(22,23)^.

Por outro lado, adultos mais velhos sem perda auditiva, com boa habilidade de processamento auditivo temporal e com capacidade reduzida de memória de trabalho e de velocidade de processamento apresentaram piores desempenho de percepção de fala no ruído quando comparados aos mais jovens. Os autores não atribuíram tal achado somente ao declínio do aspecto cognitivo relacionado à idade^(24)^, mas destacaram a variabilidade interindividual e o *status* auditivo. Diante disso, percebe-se que o comprometimento na habilidade de percepção de fala no ruído é impactado por outros fatores, principalmente, quando a população é idosa.

Tal contradição é evidenciada, também, nos estudos de revisão da literatura. Foram analisados 20 estudos e encontrou-se um link entre a percepção de fala com ruído competitivo e a cognição em diferentes indivíduos, sem e com perda auditiva (usuários de aparelho auditivo). Porém, nenhum teste cognitivo apresentou resultado significativo considerando todas as variáveis estudadas. Assim, os resultados dessa revisão evidenciaram inconsistências entre as habilidades cognitivas avaliadas e os testes de percepção de fala no ruído competitivo. Por exemplo, houve constatação da associação apenas entre o desempenho da memória de trabalho e o quociente de inteligência (QI) nos testes de fala com ruído competitivo^(25)^.

Em uma revisão com metanálise não foi observado diferença significativa entre a memória de trabalho de jovens adultos no teste de sentença na presença de ruído competitivo, em 16 estudos avaliados. Concluíram que a memória de trabalho apresenta pouca contribuição nas diferenças individuais da percepção de fala no ruído competitivo de jovens ouvintes, porém, observou-se que os estudos inseridos na revisão apresentaram uma amostra pequena e um intervalo de confiança amplo^(15)^.

Na revisão sistemática com metanálise, Dryden et al.^(8)^ analisaram 25 pesquisas. Verificaram uma associação geral fraca (*r* = 0,31) do desempenho cognitivo (atenção, memória, função executiva, QI e velocidade processamento) com a habilidade de percepção de fala no ruído em adultos ouvintes e com perda auditiva de grau leve a moderado. Ainda observaram heterogeneidade entre as metodologias dos estudos, e uma grande variabilidade nesta associação dependendo da função cognitiva, do teste de percepção de fala e do ruído mascarador avaliado^(8)^.

Especificamente com relação aos resultados do HINT-Brasil na apresentação binaural, observou-se que a população idosa com risco de alteração cognitiva apresentou melhor desempenho na condição ruído à esquerda (Tabela 1). Diante desse resultado, é importante compreender os mecanismos funcionais que ocorrem em cada condição de escuta do HINT-Brasil.

A condição do HINT-Brasil com ruído à esquerda é de escuta mais favorável quando comparada às condições do HINT-Brasil com ruído frontal e ruído à direita^(6)^. Ao analisar comparativamente cada uma delas se observa que, na condição ruído frontal a escuta é mais desfavorável, pois o ruído é apresentado na mesma intensidade que o sinal de fala bilateralmente. Há relatos de que na condição de sinal mais desafiadora, o indivíduo utiliza-se das habilidades cognitivas superiores como memória de trabalho, controle de atenção e velocidade de processamento no desempenho da percepção de fala no ruído (processamento top-down)^(6)^. Contudo, no presente estudo, os indivíduos com risco de alteração cognitiva (GII) não demonstraram pior desempenho no teste na comparação com o GI (Tabela 1).

Na condição HINT-Brasil à direita, o ruído é apresentado mais intenso na orelha direita (*unshadowed noise*) e mais fraco na orelha esquerda (*head-shadowed noise*), enquanto as sentenças são apresentadas na mesma intensidade em ambas orelhas. Para os idosos esta condição também pode representar um grau de dificuldade considerável, uma vez que, apesar da relação F/R ser mais positiva em relação à orelha direita, há desvantagem na percepção de fala da orelha esquerda com o aumento da idade^(26)^. Com o envelhecimento são observadas mudanças morfológicas nos córtices cerebrais, com maior deterioramento do hemisfério direito, além de degeneração do corpo caloso, estrutura do sistema nervoso responsável por transferir as informações entre um hemisfério e outro, o que provoca um declínio na transmissão inter-hemisférica entre as orelhas com o aumento da idade^(26,27)^. No presente estudo, o desempenho entre os grupos foi semelhante nesta condição do teste, ou seja, não houve impacto significativo do declínio cognitivo no desempenho dos indivíduos do grupo GII.

Na condição do HINT-Brasil à esquerda o sinal de fala é apresentado na mesma intensidade na orelha contralateral (direita), porém com intensidade do ruído competitivo mais forte na orelha esquerda (*unshadowed noise*) e o ruído à direita mais fraco (*head-shadowed*). Desta forma, a percepção da fala é mais favorável, uma vez que a relação F/R é maior na orelha direita. No presente estudo, para os idosos com risco de alteração cognitiva, esta condição foi a menos desafiadora no teste, portanto, o desempenho foi melhor. A vantagem da orelha direita, com ou sem ruído competitivo, é o esperada no processamento normal da fala, como relatado em estudos^(26,27)^. Contudo, o resultado encontrado no presente estudo enfatiza que mesmo diante do declínio cognitivo, a atenção no processo de reabilitação relativo à percepção da fala no ruído, principalmente com a adaptação do aparelho auditivo, deve continuar voltada à orelha direita, devido a vantagem da mesma, caso não seja viável a adaptação binaural do aparelho auditivo^(27)^.

A partir dos resultados apresentados na Tabela 2, é possível verificar que existiu diferença entre os grupos com relação à escolaridade, o que revelou possível interferência dessa condição na percepção de fala no ruído, uma vez que o desempenho no HINT-Brasil foi diferente entre os grupos (Tabela 1). O GII (com risco de alterações cognitivas) apresentou piores níveis de escolaridade. Porém, no estudo, o nível de escolaridade não apresentou relação com o teste HINT-Brasil no grupo de idosos com risco de alteração cognitiva. Já no grupo sem risco de alteração cognitiva houve correlação positiva moderada, na condição ruído à esquerda do HINT-Brasil. Assim, observou-se que quanto maior foi o nível educacional dos idosos sem alteração da função cognitiva menor a relação F/R. De acordo com estudo prévio, para um aumento de um ano na escolaridade, a relação S/R diminui, em média, 0,40 dB^(5)^.

Ressalta-se que apesar da percepção de fala no ruído ser a variável dependente neste estudo, existe relação entre fatores educacionais e status cognitivo^(16,17)^, ou seja, a cognição é dependente desta variável qualitativa. Assim, entende-se que ao realizar o agrupamento a partir dos resultados do teste WAIS-III, havia a expectativa de que no grupo sem risco para alterações cognitivas seriam encontrados os idosos com maior nível de escolaridade. Os dados apresentados na Tabela 2 confirmam tal evidência.

Com relação a variável idade verificou-se correlação moderada no GII (risco de alteração cognitiva) somente na condição ruído frontal do HINT-Brasil, ou seja, quanto maior a idade pior foi o desempenho nesta condição (Tabela 3). O aumento da idade é um fator agravante na percepção de fala na presença de ruído competitivo^(6,25)^. Contudo, a variável idade não interferiu nas demais condições do HINT-Brasil. Diante desse resultado, ressalta-se que dependendo da condição do teste de fala no ruído, ou seja, da lateralidade de apresentação da fala com ruído competitivo, poderá não evidenciar o prejuízo com o avanço da idade. Além disso, considera-se que outros fatores, além do aumento da idade, devem interferir na percepção de fala com ruído competitivo, como a variabilidade interindividual^(24)^.

Observou-se correlação fraca da variável idade com o desempenho de algumas provas do WAIS-III, como vocabulário, aritmética, compreensão, e quociente de inteligência verbal para os idosos do GI. Este achado evidencia que idosos sem risco de alteração cognitiva podem apresentar resultados piores em provas específicas do teste cognitivo influenciados pelo avanço da idade^(7)^, mas ainda com habilidades cognitivas preservadas que os diferenciam com relação ao status cognitivo. Por outro lado, as idades absolutas não influenciaram o resultado em nenhuma prova específica do teste dos idosos do GII. Diante destes achados relacionados aos grupos do estudo, pode-se perceber que quando há o comprometimento cognitivo instalado, a idade não é a variável dependente, ou seja, apesar de haver um declínio cognitivo com o avanço da idade, este poderá acontecer por outros fatores, como experiência de vida, prática de atividade física, nível de escolaridade, fatores socioeconômicos, qualidade de sono, privação sensorial, e o hábito de alimentação saudável^(16,17)^.

Importante ressaltar que todos os participantes tinham perda auditiva (Figura 1), e ao classificar os idosos nos grupos I e II a partir dos resultados do WAIS-III, observou-se uma distribuição equilibrada relativa ao grau de perda auditiva entre os grupos, ou seja, 33,3% dos indivíduos com perda auditiva sensorioneural leve e 66,7% com perda auditiva sensorioneural moderada em cada grupo. Em ambos os grupos houve correlação positiva altamente significante entre o grau da perda auditiva e a percepção de fala no ruído (Tabela 3), ou seja, quanto maior o grau da perda auditiva, maior a relação F/R demonstrando pior desempenho na percepção da fala quando o ruído estava mais elevado, como já reportado na literatura^(14)^.

Assim, independente de apresentar risco ou não de déficit da função cognitiva, quanto maior o grau da perda auditiva do idoso, maior foi a dificuldade em ambientes de escuta difíceis (Tabela 3). Existe uma lacuna no conhecimento sobre a relação entre o grau da perda auditiva e a cognição, relatada em estudos recentes^(12,13,28,29)^ No presente estudo, o grau de perda auditiva leve ou moderado não foi determinante para classificação dos participantes no GI ou GII.

Ressalta-se que neste estudo a habilidade de percepção de fala na presença do ruído competitivo sofreu influência do grau da perda auditiva e do nível de escolaridade. Contudo, não foi possível confirmar a interferência dos recursos cognitivos no teste HINT-Brasil, portanto, a hipótese do estudo não se confirmou.

Por fim, diante de todas as considerações relatadas no presente estudo e as evidências científicas apontadas na literatura, faltam indícios consistentes para entender se as dificuldades no desempenho da percepção de fala no ruído são pelo deterioramento das funções cognitivas relacionada à idade em um domínio específico, como a memória de trabalho^(25)^ pela perda auditiva relacionada à idade^(11,30)^ e/ou outro comprometimento relacionado ao envelhecimento^(12,23,24)^.

Em relação a limitação do estudo, utilizou-se os métodos estatísticos apropriados para o tamanho da amostra, possibilitando melhor compreensão da incerteza e da variabilidade dos dados, e confirma que os resultados encontrados sejam válidos. Contudo, houve um número pequeno de idosos com risco de alteração cognitiva, o que pode ter sido uma fragilidade à pesquisa.

## CONCLUSÃO

O declínio das habilidades cognitivas verbais não afetou a percepção da fala no ruído em idosos com perda auditiva. A habilidade de percepção de fala sofreu interferência das variáveis grau da perda auditiva e nível de escolaridade tanto nos idosos com risco de alteração cognitiva como naqueles sem risco. Por fim, o nível educacional interferiu no desempenho da função cognitiva.
